# Transcriptome analysis of follicular granulosa cells in young women with diminished ovarian reserve

**DOI:** 10.1186/s12958-026-01589-y

**Published:** 2026-07-15

**Authors:** Peizhe Tian, Yanfang Lu, Lei Zhang, Huanhuan Chen, Chenyue Dong, Cuilian Zhang

**Affiliations:** 1 Reproductive Medicine Center, People’s Hospital of Zhengzhou University, Henan Provincial People’s Hospital, Zhengzhou, Henan Province 450000 China; 2 Department of Nephrology, Henan Provincial Key Laboratory of Kidney Disease Innovation and Translation, Henan International Joint Laboratory of Kidney Disease and Microenvironment, Henan Provincial Clinical Research Center for Kidney Disease, Henan Provincial People’s Hospital, People’s Hospital of Zhengzhou University, Zhengzhou, 450003 China

**Keywords:** Diminished ovarian reserve, Granulosa cells, MicroRNA, Long non-coding RNA, Competing endogenous RNA, Transcriptome

## Abstract

**Background:**

Diminished ovarian reserve (DOR) is defined as the decline in both quantity and quality of oocytes in the ovarian cortex. In recent years, DOR has shown an increasing trend among young women. However, the specific etiology and detailed pathogenesis in this population remain unclear. Granulosa cells (GCs) play crucial roles in oocyte growth, development, energy absorption, and transcriptional regulation. This study aimed to investigate the differential expression profiles of microRNAs (miRNAs), long non-coding RNAs (lncRNAs), and messenger RNAs (mRNAs) in GCs from young women with DOR, and to explore the potential molecular mechanisms through competing endogenous RNA (ceRNA) network analysis.

**Methods:**

A total of 80 young women (≤ 35 years) undergoing in vitro fertilization-embryo transfer (IVF-ET) were prospectively enrolled between April 2024 and December 2024, including 40 with normal ovarian reserve (NOR) and 40 with DOR. Granulosa cells were collected during oocyte retrieval. High-throughput sequencing was performed to identify differentially expressed miRNAs, lncRNAs, and mRNAs. Bioinformatics analysis including Gene Ontology (GO) and Kyoto Encyclopedia of Genes and Genomes (KEGG) pathway enrichment was conducted. The ceRNA regulatory network was constructed using Cytoscape. Quantitative real-time PCR (qRT-PCR) was used to validate the sequencing results.

**Results:**

Compared with the NOR group, young women with DOR showed significantly higher basal follicle-stimulating hormone (FSH) levels, and significantly lower anti-Müllerian hormone (AMH) levels and antral follicle count (AFC) (all P < 0.05). The DOR group also had significantly lower numbers of retrieved oocytes, metaphase II oocytes, two-pronuclear zygotes, and day 3 available embryos (all P < 0.05). A total of 36 differentially expressed miRNAs (5 upregulated, 31 downregulated), 102 differentially expressed lncRNAs (55 upregulated, 47 downregulated), and 234 differentially expressed mRNAs (69 upregulated, 165 downregulated) were identified. Functional enrichment analysis revealed that these differentially expressed RNAs were mainly involved in fatty acid metabolism, AKT-mTOR signaling pathway, apoptosis, DNA damage and repair, and steroidogenesis. Bioinformatics analysis predicted a ceRNA network containing 20 lncRNA-mRNA pairs, 108 lncRNA-miRNA pairs, and 89 miRNA-mRNA pairs. Two potential ceRNA axes were identified by qRT-PCR-based expression validation: lncRNA MSTRG.39605.16/miR-143-5p/FADS1 and lncRNA NONHSAT208904.1/miR-199b-3p/ACP3, both associated with fatty acid metabolism regulation. However, direct molecular interactions within these axes remain to be functionally validated.”

**Conclusions:**

Transcriptional alterations in granulosa cells from young women with DOR are primarily enriched in fatty acid metabolism, AKT-mTOR signaling, and apoptosis pathways. The predicted ceRNA networks represent hypothetical regulatory mechanisms that may potentially be involved in DOR pathogenesis through modulation of fatty acid metabolic processes, pending functional validation. These findings provide candidate molecular markers and potential targets for future mechanistic studies of this condition.

**Supplementary Information:**

The online version contains supplementary material available at https://doi.org/10.1186/s12958-026-01589-y.

## Introduction

Diminished ovarian reserve (DOR) is characterized by a decline in both the quantity and quality of oocytes within the ovarian cortex, resulting in decreased female reproductive potential [1]. While ovarian reserve naturally decreases with age, many young women develop DOR due to factors beyond aging. Genetic mutations, autoimmune diseases, medical treatments, and environmental exposures have all been implicated in DOR development, suggesting that age is not the sole determinant [2]. Young women with DOR face poor assisted reproductive technology (ART) outcomes, including reduced oocyte retrieval, lower embryo quality, and decreased clinical pregnancy rates [3]. Currently available clinical markers such as basal follicle-stimulating hormone (FSH), anti-Müllerian hormone (AMH), and antral follicle count (AFC) primarily reflect follicle quantity rather than oocyte quality. Furthermore, changes in these parameters often indicate irreversible ovarian damage [4]. Therefore, elucidating the molecular mechanisms underlying DOR in young women is essential for early diagnosis and intervention.

Granulosa cells (GCs) are somatic cells intimately associated with oocytes, providing essential support for follicular development, oocyte maturation, and hormone secretion [5, 6]. Bidirectional communication between oocytes and GCs is critical for normal follicular development and oocyte competence [7]. GCs influence oocyte developmental capacity through transcriptional, protein, and metabolic regulation, and GC dysfunction has been implicated in various ovarian pathologies [8]. Recent studies have demonstrated that non-coding RNAs (ncRNAs), including microRNAs (miRNAs) and long non-coding RNAs (lncRNAs), play pivotal roles in regulating ovarian maturation, GC development, and hormone secretion [9, 10].

MiRNAs are approximately 22-nucleotide single-stranded ncRNAs that regulate gene expression post-transcriptionally by binding to the 3’-untranslated region (3’-UTR) of target mRNAs, leading to translational repression or mRNA degradation [11]. LncRNAs are transcripts longer than 200 nucleotides that function as miRNA sponges in competing endogenous RNA (ceRNA) networks, thereby modulating target gene expression [12]. The ceRNA hypothesis proposes that lncRNAs, miRNAs, and mRNAs interact through shared miRNA response elements (MREs), forming complex regulatory networks that influence various physiological and pathological processes [13]. Previous studies have identified altered miRNA and lncRNA expression profiles in GCs from patients with polycystic ovary syndrome (PCOS) [14] and advanced maternal age-related ovarian aging [15]. Notably, these studies focused on pathological conditions with distinct etiologies—PCOS represents hyperandrogenic anovulation, while age-related DOR reflects physiological senescence. In contrast, young women with DOR (≤ 35 years) represent a unique clinical phenotype: they exhibit accelerated ovarian reserve decline despite age-appropriate morphology, suggesting pathological mechanisms distinct from both PCOS and chronological aging. However, ceRNA-based transcriptome analysis in this specific population remains entirely absent from the literature.

Therefore, this study specifically focused on young women with DOR. We characterized the full transcriptome of their granulosa cells and compared them to age-matched controls with normal ovarian reserve. Using high-throughput sequencing, we identified differentially expressed miRNAs, lncRNAs, and mRNAs. We then constructed ceRNA networks and performed functional enrichment analysis. Our findings provide preliminary insights into potential molecular mechanisms that may contribute to DOR in young women, and identify candidate diagnostic biomarkers and therapeutic targets warranting further validation.

## Materials and methods

### Study design and participants

This prospective study was conducted at the Reproductive Center of People’s Hospital of Zhengzhou University (Henan Provincial People’s Hospital) between April 2024 and December 2024. The study protocol was approved by the Ethics Committee of Henan Provincial People’s Hospital and conducted in accordance with the Declaration of Helsinki. All participants provided written informed consent.

A total of 80 young women (age ≤ 35 years) undergoing their first in vitro fertilization-embryo transfer (IVF-ET) cycle were enrolled. Participants were divided into two groups based on ovarian reserve parameters: (1) Young DOR group (YD, *n* = 40): bilateral AFC < 7 and serum AMH < 1.2 ng/mL; (2) Young normal ovarian reserve group (YN, *n* = 40): bilateral AFC 7–24 and serum AMH ≥ 1.2 ng/mL. Exclusion criteria included: (1) PCOS, diabetes, thyroid dysfunction, or hyperprolactinemia; (2) severe endometriosis, endometrial abnormalities, or intrauterine adhesions; (3) male factor infertility requiring surgical sperm retrieval; (4) chromosomal abnormalities in either partner.

### Controlled ovarian stimulation and sample collection

Controlled ovarian stimulation was performed using either GnRH agonist long protocol or GnRH antagonist protocol based on individual patient characteristics(The two stimulation protocols were evenly distributed between the DOR and NOR groups). For the agonist protocol, 3.75 mg long-acting triptorelin (Yipu, Tianjin) was administered on day 2–3 of the menstrual cycle or 0.1 mg/day short-acting triptorelin (Decapeptyl, Ferring, Germany) for 14–18 days in the mid-luteal phase of the previous cycle. After pituitary downregulation, recombinant FSH (Gonal-f, Merck Serono) was initiated at 75–300 IU/day. For the antagonist protocol, recombinant FSH was started on day 2–3 of the menstrual cycle, and 0.25 mg/day cetrorelix (Cetrotide, Merck Serono) was added when the leading follicle reached 12 mm or estradiol exceeded 200 ng/L. Final oocyte maturation was triggered with hCG (4,000–10,000 IU) when at least two follicles reached ≥ 18 mm or three follicles reached ≥ 17 mm. Oocyte retrieval was performed 35–36 h later under transvaginal ultrasound guidance.

Follicular fluid was collected from the first clear, blood-free aspirate during oocyte retrieval. Granulosa cells were isolated by density gradient centrifugation using Ficoll-Paque (GE Healthcare) followed by red blood cell lysis. Cells were washed twice with phosphate-buffered saline, pelleted, and stored at -80 °C until RNA extraction.

### RNA extraction and quality control

Total RNA was extracted from GCs using TRIzol reagent (Invitrogen, USA) according to the manufacturer’s protocol. RNA concentration and purity were assessed using NanoDrop ND-2000 spectrophotometer (Thermo Fisher Scientific). RNA integrity was evaluated using Agilent 2100 Bioanalyzer (Agilent Technologies). Samples with RNA integrity number (RIN) ≥ 6.0, 28 S/18S ratio ≥ 0.7, and A260/A280 ratio between 1.8 and 2.1 were considered suitable for sequencing.

### High-throughput sequencing and data analysis

For miRNA sequencing, small RNA libraries were constructed using the NEBNext Multiplex Small RNA Library Prep Kit (New England Biolabs). Briefly, 3’ and 5’ adapters were ligated to purified total RNA, followed by reverse transcription and PCR amplification. Libraries were size-selected for 140–160 bp fragments and quantified using Qubit 2.0 Fluorometer (Life Technologies). For mRNA and lncRNA sequencing, ribosomal RNA was removed using Ribo-Zero rRNA Removal Kit (Illumina). The remaining RNA was fragmented, reverse-transcribed, and ligated with sequencing adapters. All libraries were sequenced on the Illumina NovaSeq 6000 platform (Illumina, USA) with 150 bp paired-end reads.

Raw reads were filtered to remove low-quality sequences, adapter contaminants, and reads shorter than 18 nucleotides. Clean reads were aligned to the human reference genome (GRCh38/hg38) using HISAT2 (v2.1.0) for mRNA/lncRNA and Bowtie (v1.3.0) for miRNA. Gene expression levels were quantified as fragments per kilobase of transcript per million mapped reads (FPKM) for mRNAs/lncRNAs and transcripts per million (TPM) for miRNAs. FPKM/TPM normalization was applied to account for sequencing depth and gene length differences. Batch effects were assessed by principal component analysis and inter-sample correlation; no significant batch effects were detected as all samples were processed and sequenced in a single batch.

### Identification of differentially expressed RNAs

Differential expression analysis was performed using edgeR (v3.32.1) [16]. RNAs with |log2 fold change| ≥1 and false discovery rate (FDR) < 0.05 were considered significantly differentially expressed. Principal component analysis (PCA) and Pearson correlation analysis were conducted to assess sample relationships and reproducibility. Volcano plots and heatmaps were generated using R software (v4.0.3) to visualize differential expression patterns .

### Functional enrichment analysis

Gene Ontology (GO) enrichment analysis was performed to annotate differentially expressed genes in three categories: biological process (BP), cellular component (CC), and molecular function (MF). Kyoto Encyclopedia of Genes and Genomes (KEGG) pathway analysis was conducted to identify significantly enriched signaling pathways. GO and KEGG analyses were implemented using clusterProfiler (v3.18.1) [17]. Terms with *P* < 0.05 were considered statistically significant.

### Target gene prediction and ceRNA network construction

For miRNAs, target genes were predicted using miRanda (v3.3a) and TargetScan (v7.2) algorithms [18, 19]. Only targets predicted by both tools were retained for subsequent analysis. For lncRNAs, cis-regulatory targets were defined as protein-coding genes located within 10 kb upstream or downstream, while trans-regulatory targets were predicted based on sequence complementarity using RNAplex [20].

The ceRNA network was constructed based on the following criteria: (1) miRNA and candidate ceRNAs showed negative correlation (Spearman’s rank correlation coefficient ≤-0.7); (2) candidate ceRNA pairs showed positive correlation (Pearson’s correlation coefficient ≥ 0.9); (3) hypergeometric test *P* < 0.05 for shared miRNA enrichment. The network was visualized using Cytoscape (v3.8.2) [21].

From the constructed network, two candidate ceRNA axes were prioritized for experimental validation: lncRNA MSTRG.39605.16/miR-143-5p/FADS1 and lncRNA NONHSAT208904.1/miR-199b-3p/ACP3. These axes were selected based on integrated criteria: (1) all component RNAs ranked among the top differentially expressed genes; (2) regulatory relationships met the stringent correlation and prediction thresholds described above; (3) both axes converged on fatty acid metabolism, the most consistently enriched pathway across all RNA categories; and (4) the selected miRNAs have established roles in metabolic regulation according to published literature.

### Quantitative real-time PCR validation

Selected differentially expressed miRNAs (miR-143-5p, miR-199b-3p, miR-199a-3p, miR-126-3p, miR-144-5p, miR-10b-5p), lncRNAs (MSTRG.39605.16, NONHSAT208904.1, NONHSAT177785.1, NONHSAT188349.1, NONHSAT247791.1), and mRNAs (FADS1, ACP3, DHCR7, CYP51A1, MSMO1, INSIG1) were validated by qRT-PCR using the remaining 28 samples from each group (not used for sequencing).

For miRNA quantification, total RNA was reverse-transcribed using miScript II RT Kit (Qiagen). qPCR was performed with miScript SYBR Green PCR Kit (Qiagen) on LightCycler 96 (Roche). U6 small nuclear RNA served as the internal reference. For mRNA and lncRNA quantification, cDNA was synthesized using ReverTra Ace qPCR RT Kit (Toyobo). qPCR was conducted using RealStar Green Fast Mixture (GenStar) with GAPDH as the reference gene. Relative expression levels were calculated using the 2 < sup> method [22]. All primers were synthesized by Beijing Tsingke Biotechnology (sequences available upon request).

### Statistical analysis

Statistical analyses were performed using SPSS 21.0 (IBM, USA). Continuous variables were tested for normality using the Shapiro-Wilk test. Normally distributed data were expressed as mean ± standard deviation (SD) and compared using independent samples t-test. Categorical variables were expressed as frequencies and percentages and compared using Chi-square test. Correlation analysis was performed using Pearson or Spearman correlation as appropriate. *P* < 0.05 was considered statistically significant.

## Results

### Clinical characteristics of study participants

The clinical and laboratory characteristics of the two groups are summarized in Table 1. No significant differences were observed in age, infertility duration, or body mass index (BMI) between the YD and YN groups (all *P* > 0.05). As expected, the YD group exhibited significantly higher basal FSH levels (11.25 ± 1.02 vs. 6.02 ± 1.56 U/L, *P* = 0.003) and significantly lower AMH levels (0.61 ± 0.35 vs. 4.90 ± 3.70 ng/mL, *P* < 0.001) and AFC (3.67 ± 1.54 vs. 15.24 ± 4.65, *P* < 0.001) compared to the YN group.

**Table 1:** Clinical characteristics and IVF outcomes of study participants

Characteristic	YD group (*n* = 40)	YN group (*n* = 40)	t/χ² value	*P* value
Age (years)	31.58 ± 2.63	31.13 ± 3.19	0.80	0.426
Infertility duration (years)	3.54 ± 2.60	3.10 ± 2.31	0.78	0.437
BMI (kg/m²)	23.01 ± 3.11	23.23 ± 3.06	−0.24	0.756
Basal FSH (U/L)	11.25 ± 1.02	6.02 ± 1.56	3.12	0.003
AMH (ng/mL)	0.61 ± 0.35	4.90 ± 3.70	−7.39	< 0.001
AFC (n)	3.67 ± 1.54	15.24 ± 4.65	−15.03	< 0.001
Total Gn dosage (U)	2405.92 ± 922.75	2185.00 ± 897.30	1.07	0.033
Gn duration (days)	8.42 ± 2.91	9.68 ± 3.15	−1.82	0.234
Starting Gn dose (U)	269.08 ± 57.08	200.31 ± 67.55	4.84	< 0.001
Retrieved oocytes (n)	3.24 ± 2.12	11.08 ± 5.20	−8.99	< 0.001
FORT (%)	59.31 (86/145)	60.36 (373/618)	0.05	0.817
MII oocytes (n)	2.63 ± 1.93	8.76 ± 4.94	−7.37	< 0.001
2PN zygotes (n)	1.79 ± 4.69	6.24 ± 4.16	−3.39	< 0.001
Day 3 available embryos (n)	2.10 ± 1.51	5.94 ± 3.93	−5.13	< 0.001

Values are presented as mean ± SD or percentage (ratio)

*YD* Young diminished ovarian reserve, *YN* Young normal ovarian reserve, *BMI* Body mass index, *FSH* Follicle-stimulating hormone, *AMH* Anti-Müllerian hormone,* AFC* Antral follicle count, *Gn* Gonadotropin, *FORT* Follicular output rate, *MII* Metaphase II, *2PN* Two pronuclei

Regarding ART outcomes, the YD group required significantly higher total gonadotropin dosage (2405.92 ± 922.75 vs. 2185.00 ± 897.30 U, *P* = 0.033) and starting dose (269.08 ± 57.08 vs. 200.31 ± 67.55 U, *P* < 0.001). The numbers of retrieved oocytes (3.24 ± 2.12 vs. 11.08 ± 5.20, *P* < 0.001), metaphase II oocytes (2.63 ± 1.93 vs. 8.76 ± 4.94, *P* < 0.001), two-pronuclear zygotes (1.79 ± 4.69 vs. 6.24 ± 4.16, *P* < 0.001), and day 3 available embryos (2.10 ± 1.51 vs. 5.94 ± 3.93, *P* < 0.001) were significantly lower in the YD group. No significant difference was observed in follicular output rate (FORT) between groups (*P* = 0.817).

### Differential miRNA expression profiles

**Fig. 1:**
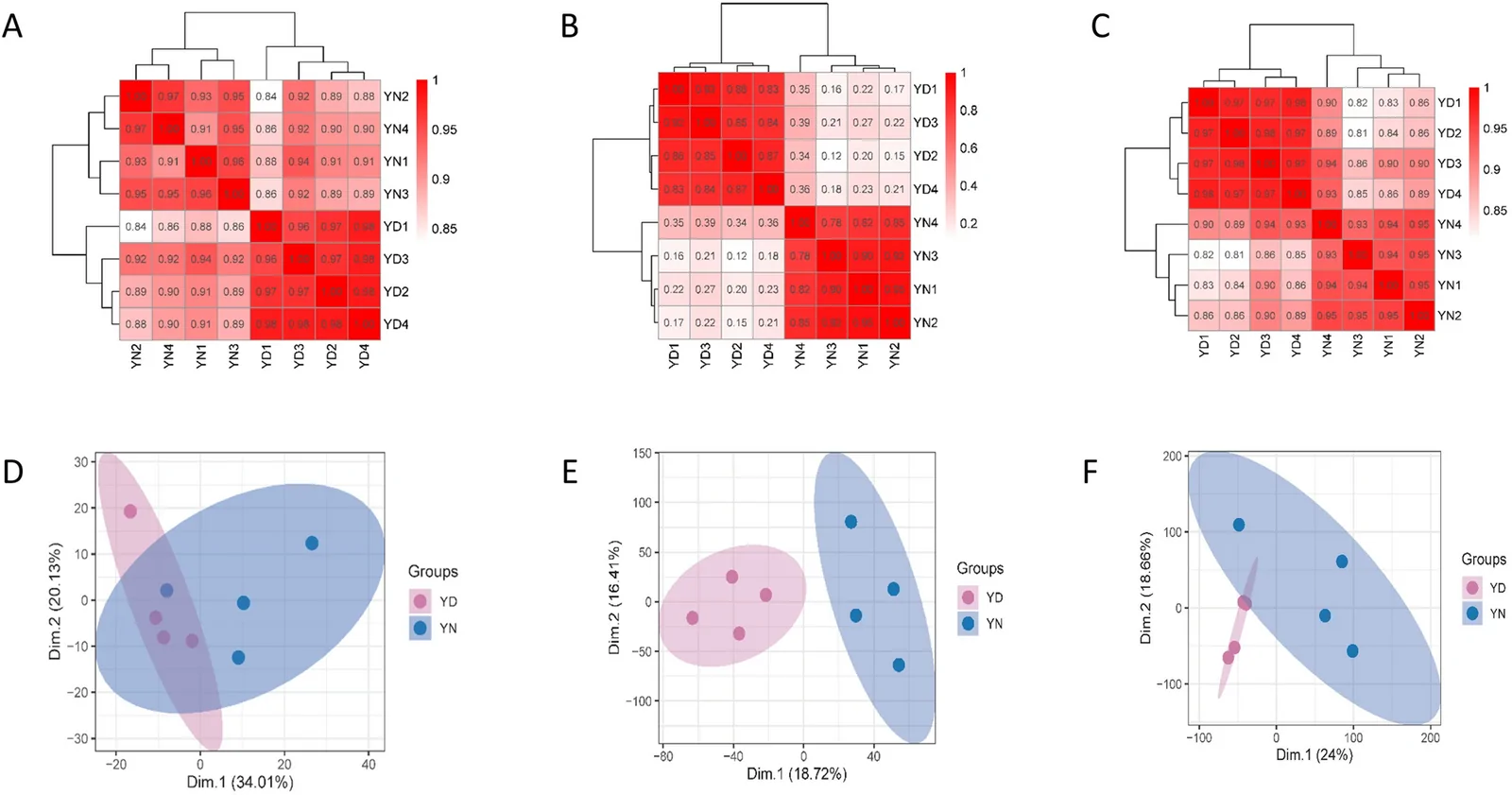
Quality control of high-throughput sequencing data. Spearman correlation analysis (upper panel, **A**-**C**) and principal component analysis (PCA, lower panel, **E**-**G**) for miRNA (**A**, **E**), lncRNA (**B**, **F**), and mRNA (**C**, **G**) expression profiles. YD: young diminished ovarian reserve; YN: young normal ovarian reserve. High inter-sample correlations and clear group separation confirm data reliability

High-throughput sequencing was performed on 8 granulosa cell samples (4 from each group). All samples were processed and sequenced in a single batch. Batch effects were assessed by principal component analysis and inter-sample correlation; no significant batch effects were detected, as evidenced by PCA clustering driven by biological group rather than technical factors (Fig. 1D-F). Quality control metrics confirmed excellent RNA integrity with RIN values ≥ 6.0 and 28 S/18S ratios ≥ 0.7. Pearson correlation analysis and PCA demonstrated high inter-sample consistency within groups and clear separation between groups (Fig. 1A, D), indicating reliable sequencing data.

**Fig. 2:**
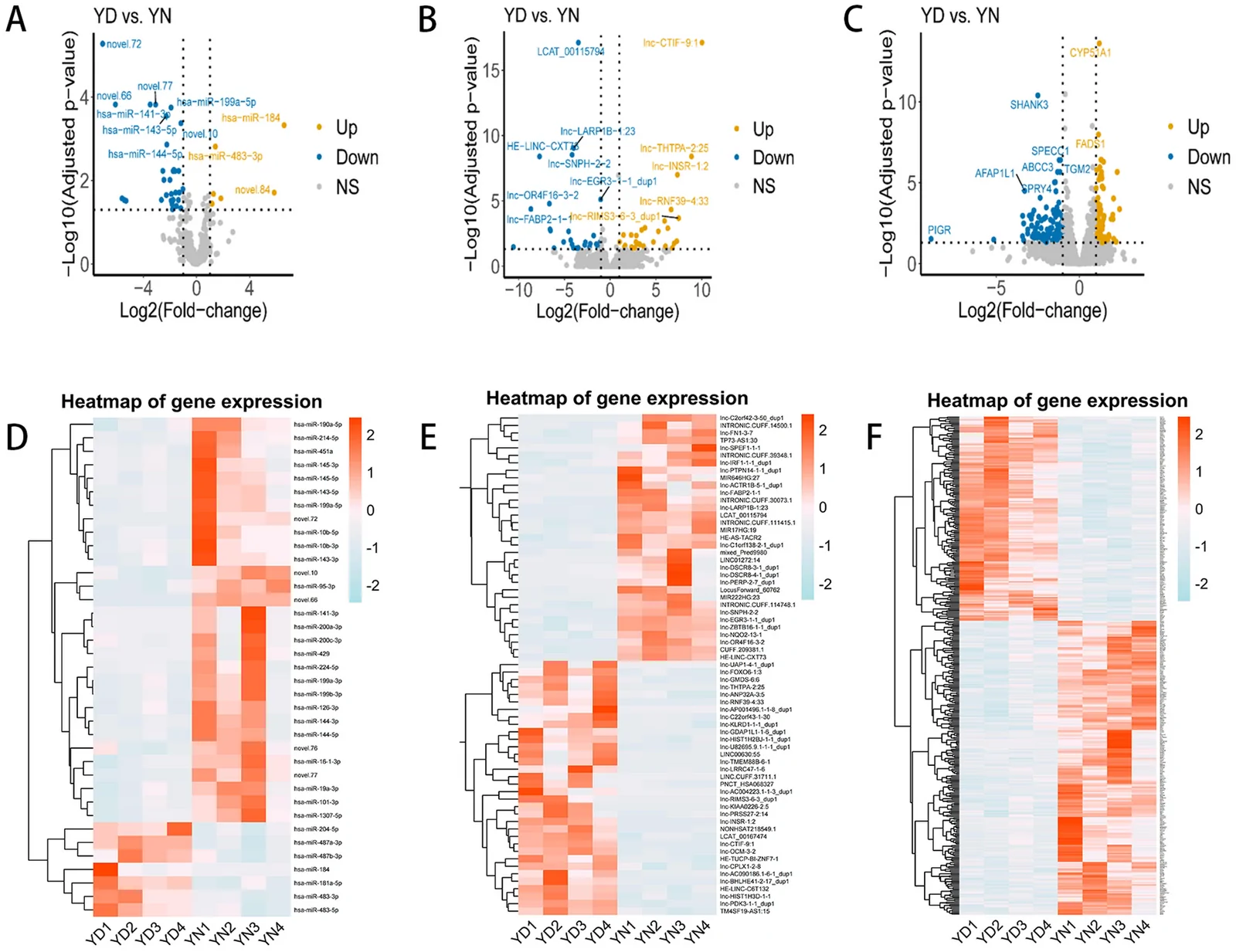
Differential expression analysis of miRNAs, lncRNAs, and mRNAs in granulosa cells from young women with diminished ovarian reserve. **A**-**C** Volcano plots showing differentially expressed miRNAs (**A**), lncRNAs (**B**), and mRNAs (**C**). Yellow/Orange indicates upregulated RNAs, blue indicates downregulated RNAs. **D**-**F** Heatmaps of differentially expressed miRNAs (**D**), lncRNAs (**E**), and mRNAs (**F**). Red indicates high expression, blue indicates low expression

A total of 36 differentially expressed miRNAs were identified between YD and YN groups, comprising 5 upregulated and 31 downregulated miRNAs (Fig. 2A, D). Hierarchical clustering analysis revealed distinct miRNA expression patterns between the two groups. Sequencing-based validation confirmed significantly lower expression of all six selected miRNAs in YD group (p < 0.01) (Fig. s1).

**Fig. 3:**
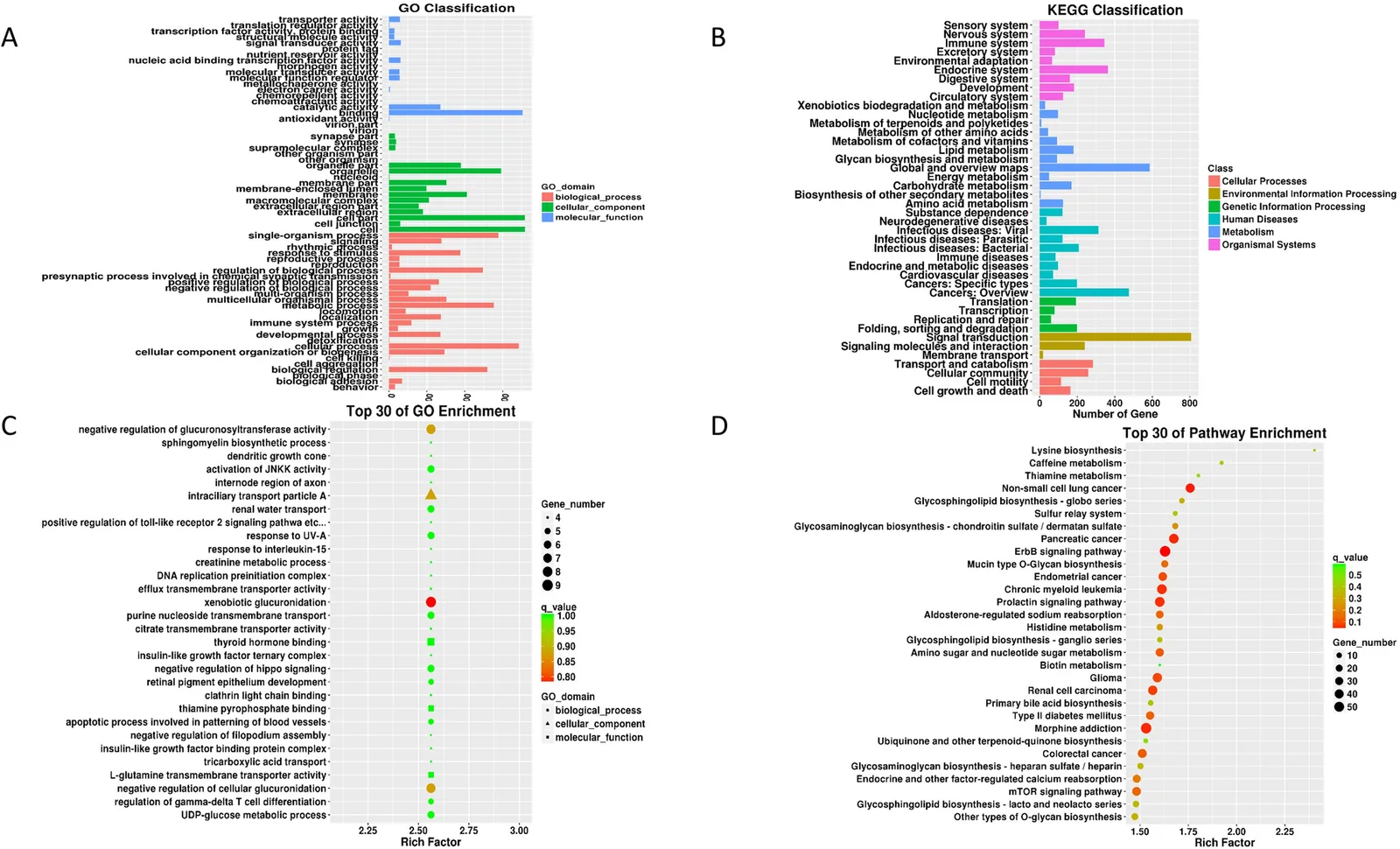
Functional enrichment analysis of differentially expressed miRNA targets. **A** GO functional classification in biological process (BP), cellular component (CC), and molecular function (MF) categories. **B** KEGG pathway classification. **C** GO enrichment scatter plot (top 30 terms). **D** KEGG enrichment scatter plot (top 30 pathways). Rich Factor indicates enrichment degree; dot size represents gene count; color intensity represents q-value significance

Target prediction identified 8,642 potential target genes for the differentially expressed miRNAs. GO analysis showed that these targets were distributed across biological process, cellular component, and molecular function categories (Fig. 3A). KEGG pathway classification revealed involvement in multiple signaling pathways including metabolism, genetic information processing, and organismal systems (Fig.3B). Further GO enrichment analysis revealed significant enrichment in biological processes including inflammatory response, apoptotic process, DNA replication, and cellular glucuronidation (Fig. 3C). KEGG pathway enrichment analysis demonstrated significant enrichment in ErbB signaling pathway, mTOR signaling pathway, endometrial cancer, prolactin signaling pathway, and histidine metabolism (Fig. 3D). These pathways are known to be involved in cell proliferation, survival, and metabolic regulation.

### Differential lncRNA expression profiles

A total of 102 differentially expressed lncRNAs were identified, including 55 upregulated and 47 downregulated transcripts in the YD group (Fig. 2B, E). PCA and correlation analysis confirmed good sample quality and group separation (Fig. 1B, E). Sequencing-based validation confirmed the differential expression patterns of all five selected lncRNAs (*p* < 0.05) (Fig. s2).

**Fig. 4:**
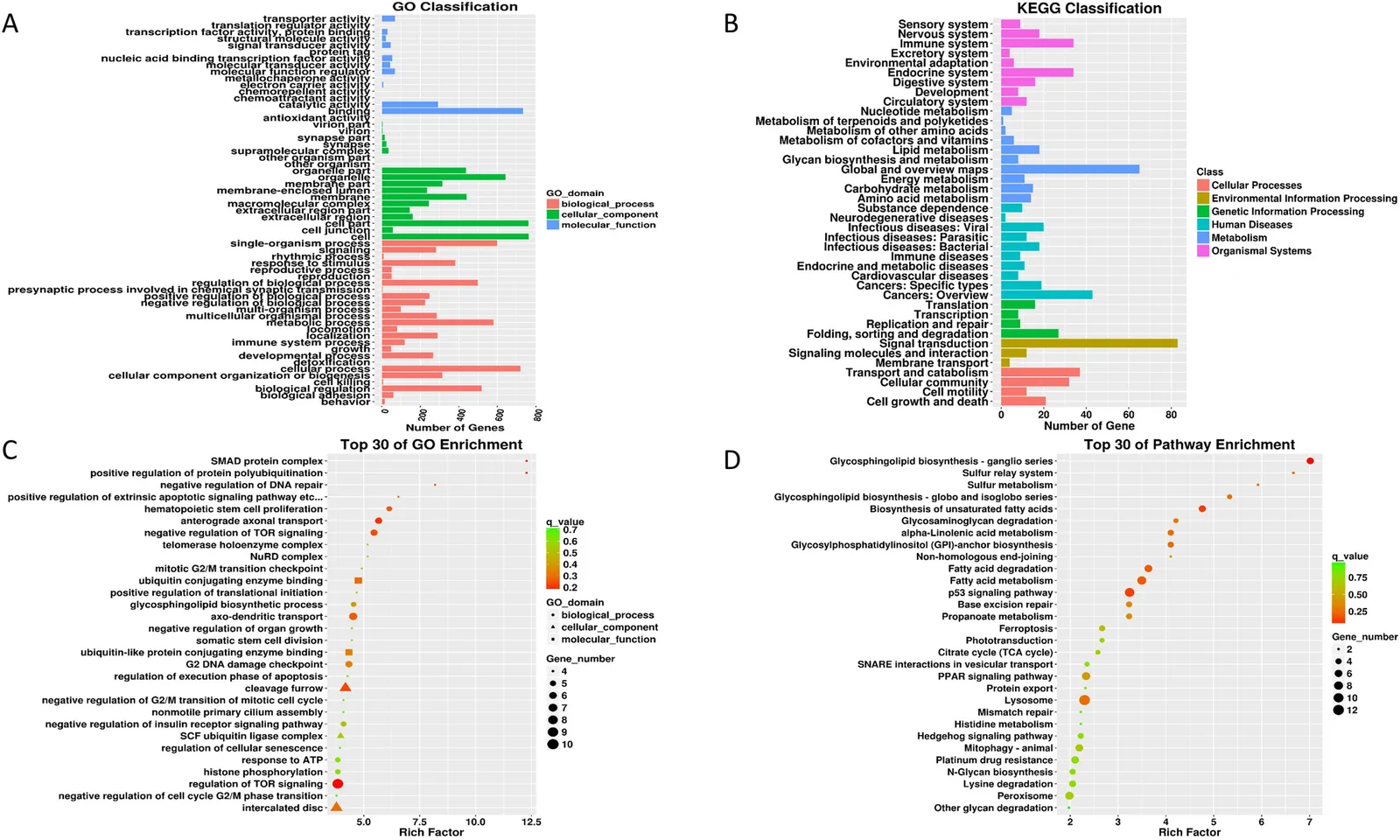
Functional enrichment analysis of differentially expressed lncRNA targets. **A** GO functional classification in BP, CC, and MF categories. **B** KEGG pathway classification. **C** GO enrichment scatter plot. **D** KEGG enrichment scatter plot. Rich Factor indicates enrichment degree; dot size represents gene count; color intensity represents *q*-value significance

Target gene prediction identified 67 cis-regulatory and 855 trans-regulatory target genes. GO classification showed distribution across all three GO domains (Fig. 4A), while KEGG classification indicated involvement in metabolism, genetic information processing, and cellular processes (Fig. 4B). Further GO enrichment analysis revealed enrichment in biological processes such as positive regulation of protein polyubiquitination, negative regulation of DNA repair, positive regulation of extrinsic apoptotic signaling pathway, and hematopoietic stem cell proliferation (Fig. 4C). Cellular component enrichment included SMAD protein complex, telomerase holoenzyme complex, and NuRD complex. KEGG pathway enrichment analysis identified significant enrichment in fatty acid metabolism, p53 signaling pathway, fatty acid degradation, base excision repair, and ferroptosis (Fig. 4D). Notably, fatty acid metabolism-related pathways were prominently represented, suggesting metabolic dysregulation in GCs from young women with DOR.

### Differential mRNA expression profiles

Transcriptome analysis identified 234 differentially expressed mRNAs (69 upregulated, 165 downregulated) in the YD group (Fig. 2C, F). The top 30 differentially expressed genes included AFAP1L1, PIGR, ITGB4, KRT19, CX3CL1, and genes involved in lipid metabolism and steroidogenesis (Table 2). Sequencing-based validation confirmed significantly higher expression of all six selected mRNAs in YD group (*p* < 0.001) (Fig. s3).

**Table 2:** Top 30 differentially expressed mRNAs in granulosa cells from young women with DOR

gene ID	gene name	gene ID	gene name
ENSG00000157510	AFAP1L1	ENSG00000267107	PCAT19
ENSG00000162896	PIGR	ENSG00000163283	ALPP
ENSG00000132470	ITGB4	ENSG00000268083	AC008982.1
ENSG00000147113	DIPK2B	ENSG00000123843	C4BPB
ENSG00000261915	AC026954.2	ENSG00000178175	ZNF366
ENSG00000248672	LY75-CD302	ENSG00000163435	ELF3
ENSG00000171345	KRT19	ENSG00000007908	SELE
ENSG00000006210	CX3CL1	ENSG00000204961	PCDHA9
ENSG00000198542	ITGBL1	ENSG00000101443	WFDC2
ENSG00000243509	TNFRSF6B	ENSG00000151322	NPAS3
ENSG00000012223	LTF	ENSG00000135480	KRT7
ENSG00000062038	CDH3	ENSG00000205038	PKHD1L1
ENSG00000236156	CHCHD4P3	ENSG00000077274	CAPN6
ENSG00000117983	MUC5B	ENSG00000254806	SYS1-DBNDD2
ENSG00000141052	MYOCD	ENSG00000135373	EHF

**Fig. 5:**
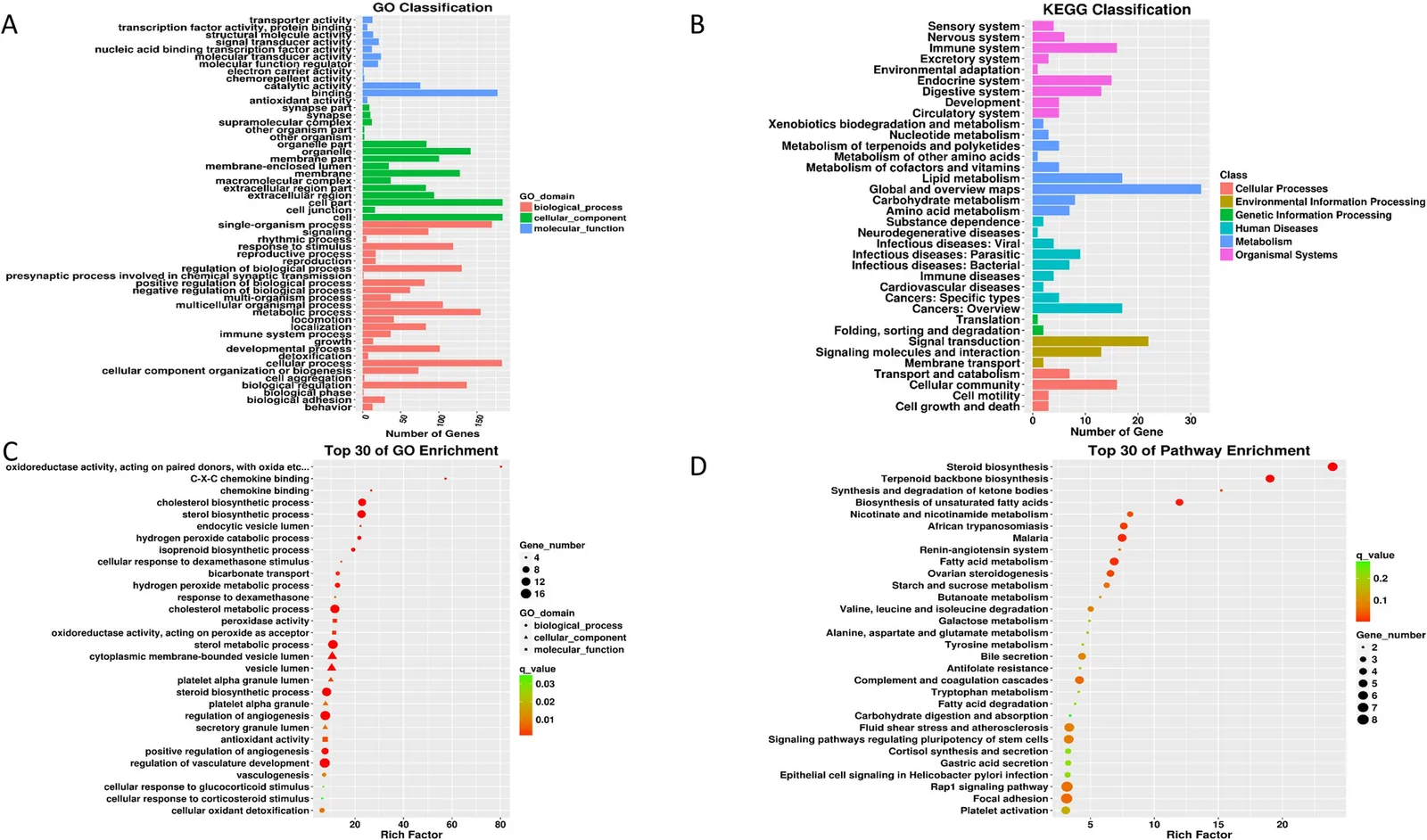
Functional enrichment analysis of differentially expressed mRNAs. **A** GO functional classification in BP, CC, and MF categories. **B** KEGG pathway classification. **C** GO enrichment scatter plot. **D** KEGG enrichment scatter plot. Rich Factor indicates enrichment degree; dot size represents gene count; color intensity represents q-value significance

GO classification of differentially expressed mRNAs showed distribution across biological process, cellular component, and molecular function categories (Fig. 5A). KEGG pathway classification revealed predominant involvement in metabolic pathways (Fig. 5B). Further GO enrichment analysis revealed significant enrichment in cholesterol biosynthetic process, sterol biosynthetic process, hydrogen peroxide catabolic process, isoprenoid biosynthetic process, and response to dexamethasone (Fig. 5C). Cellular component analysis showed enrichment in endocytic vesicle lumen, platelet alpha granule lumen, and extracellular matrix. Molecular function enrichment included oxidoreductase activity, chemokine binding, peroxidase activity, and iron ion binding. KEGG pathway enrichment analysis demonstrated significant enrichment in steroid biosynthesis, terpenoid backbone biosynthesis, synthesis and degradation of ketone bodies, biosynthesis of unsaturated fatty acids, and fatty acid metabolism (Fig. 5D). These results consistently pointed toward metabolic alterations, particularly in lipid and steroid metabolism pathways.

### ceRNA network construction and validation

**Fig. 6:**
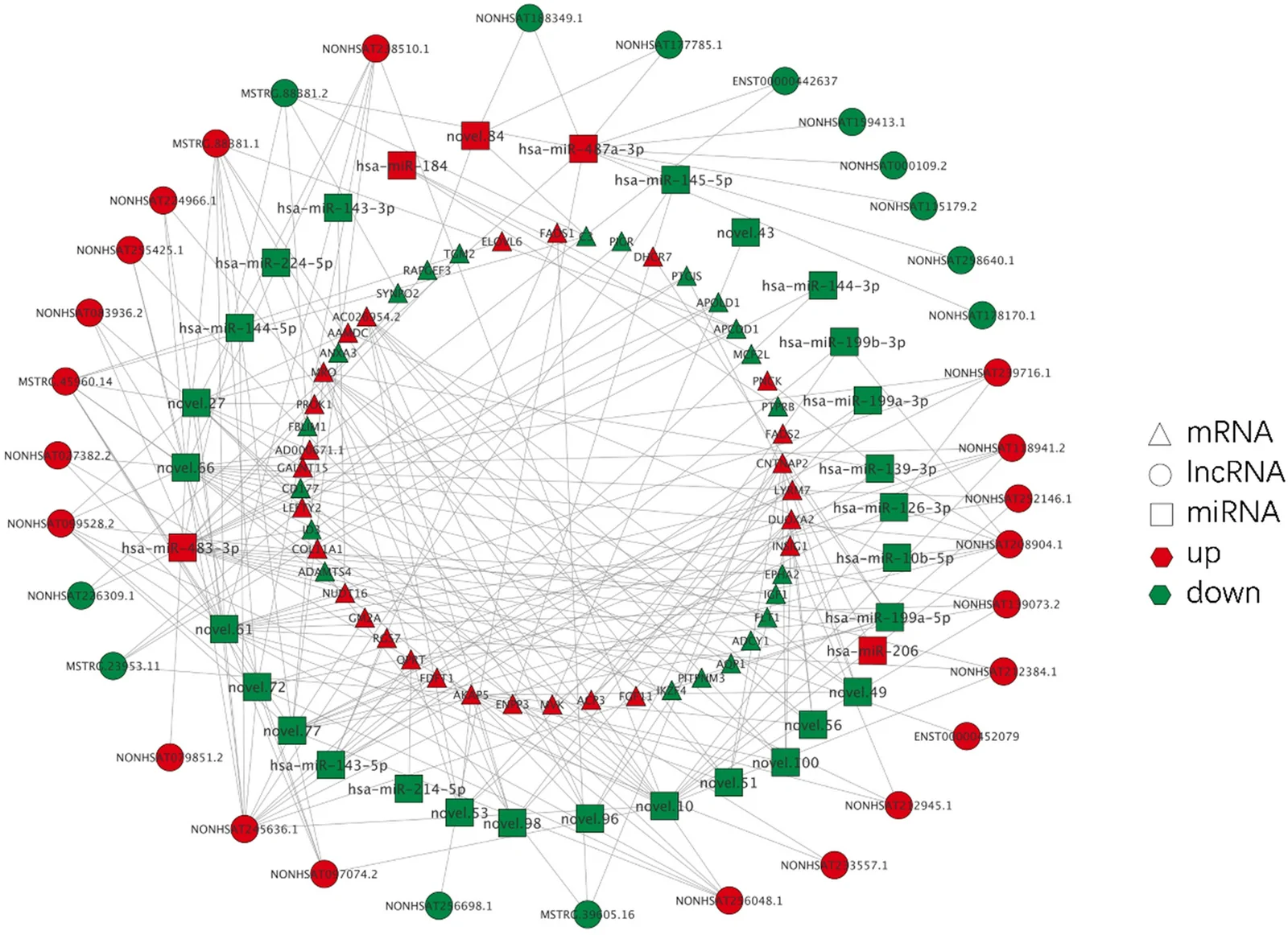
Competing endogenous RNA (ceRNA) regulatory network in young women with diminished ovarian reserve. The integrated lncRNA-miRNA-mRNA network was constructed using Cytoscape based on differentially expressed RNAs. Triangular nodes represent mRNAs, circular nodes represent lncRNAs, and square nodes represent miRNAs. Red indicates upregulation in YD group, green indicates downregulation in YD group. Edges represent predicted regulatory relationships. The network comprises 20 lncRNA-mRNA pairs, 108 lncRNA-miRNA pairs, and 89 miRNA-mRNA interactions

Based on the ceRNA hypothesis, we constructed an integrated lncRNA-miRNA-mRNA regulatory network using Cytoscape (Fig. 6). The network comprised 20 lncRNA-mRNA pairs, 108 lncRNA-miRNA pairs, and 89 miRNA-mRNA interactions. Two key ceRNA regulatory axes were identified and selected for experimental validation: (1) lncRNA MSTRG.39605.16/miR-143-5p/FADS1; (2) lncRNA NONHSAT208904.1/miR-199b-3p/ACP3. Both axes were predicted to be involved in fatty acid metabolism regulation.

**Fig. 7:**
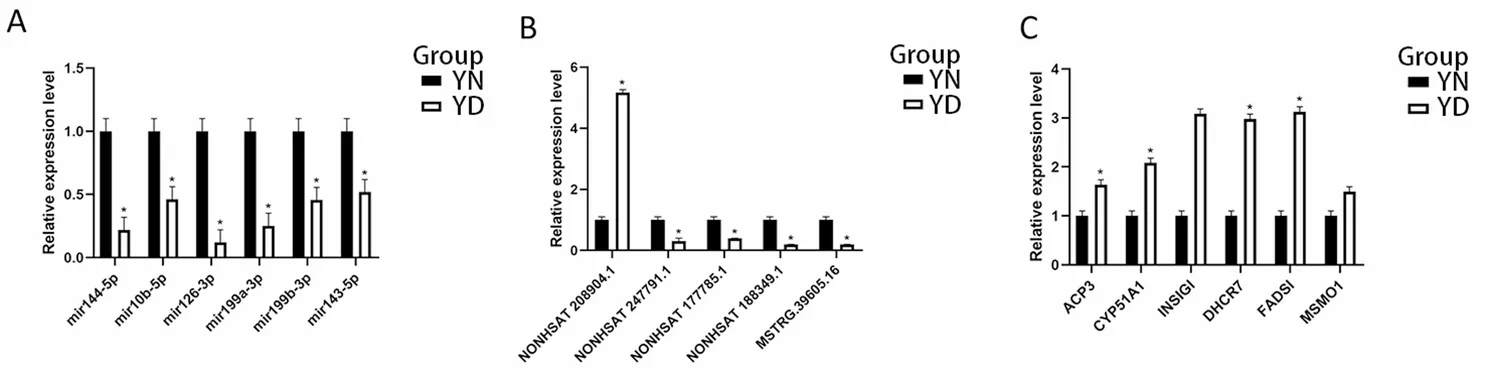
Validation of differentially expressed RNAs by qRT-PCR. **A** Relative expression levels of six miRNAs in granulosa cells from young DOR (YD, *n* = 28) and normal ovarian reserve (YN, *n* = 28) groups. **B** Relative expression levels of five lncRNAs. **C** Relative expression levels of six mRNAs. *P < 0.05, **P < 0.01 vs. YN group

qRT-PCR validation was performed using independent granulosa cell samples (*n* = 28 per group). All six selected miRNAs (miR-143-5p, miR-199b-3p, miR-199a-3p, miR-126-3p, miR-144-5p, miR-10b-5p) showed downregulation in the YD group, consistent with sequencing results (Fig. 7A). Among the five selected lncRNAs, NONHSAT208904.1 was upregulated while MSTRG.39605.16, NONHSAT177785.1, NONHSAT188349.1, and NONHSAT247791.1 were downregulated in the YD group, all consistent with sequencing data (Fig. 7B). For mRNAs, ACP3, DHCR7, CYP51A1, MSMO1, INSIG1, and FADS1 were upregulated in the YD group (Fig. 7C). These validation results confirmed the reliability of our sequencing data (Fig. S1- s3).

## Discussion

This study represents the comprehensive transcriptome analysis of granulosa cells from young women with diminished ovarian reserve, utilizing high-throughput sequencing to profile miRNA, lncRNA, and mRNA expression patterns. Our findings reveal significant transcriptional alterations predominantly enriched in fatty acid metabolism, AKT-mTOR signaling, apoptosis, DNA damage repair, and steroidogenesis pathways. Furthermore, we computationally predicted ceRNA regulatory networks and validated the differential expression patterns of their components, which represent hypothetical mechanisms that may potentially be involved in DOR pathogenesis through modulation of fatty acid metabolic processes, pending direct functional validation.

Our clinical data confirmed that young women with DOR (≤ 35 years) exhibited significantly compromised ovarian response to stimulation and poor ART outcomes despite having age-appropriate morphological characteristics. The YD group required higher gonadotropin dosages yet produced fewer oocytes and embryos of potentially lower quality, consistent with previous reports [23]. These findings underscore that chronological age alone is insufficient to predict ovarian reserve and highlight the need for molecular markers that can identify young women at risk for accelerated ovarian aging.

The differentially expressed ncRNAs identified in our study were functionally enriched in several pathways critical for ovarian function. Notably, lipid metabolic processes—particularly fatty acid metabolism—emerged as a consistently enriched pathway across all three RNA categories (miRNAs, lncRNAs, and mRNAs). Fatty acids serve as essential energy substrates for oocyte maturation and early embryonic development [24–26]. While moderate fatty acid oxidation is beneficial, excessive saturated free fatty acids can induce endoplasmic reticulum stress and apoptosis in granulosa cells and cumulus-oocyte complexes, impairing oocyte developmental competence [27]. Our findings suggest that dysregulated lipid metabolism in GCs may contribute to DOR pathogenesis in young women, warranting further investigation into the precise mechanisms linking these metabolic disturbances to ovarian dysfunction.

The mTOR signaling pathway, enriched in differential miRNA target analysis, plays a central role in regulating cell growth, proliferation, and metabolism [28]. Previous studies have demonstrated that mTOR signaling is essential for primordial follicle activation, granulosa cell proliferation, and oocyte meiotic progression [29]. Dysregulated mTOR activity has been associated with premature activation of the primordial follicle pool and accelerated ovarian reserve depletion [30]. Our identification of miRNAs targeting mTOR pathway components suggests that post-transcriptional regulation may contribute to pathway dysregulation in young DOR patients.

DNA damage repair capacity is critical for maintaining oocyte quality and ovarian reserve [31]. Accumulation of DNA double-strand breaks (DSBs) in oocytes and granulosa cells, coupled with diminished repair capacity, has been implicated in ovarian aging and DOR [32]. Our finding that lncRNA targets were enriched in DNA damage repair pathways suggests that epigenetic regulation of DNA repair mechanisms may be compromised in young women with DOR, potentially accelerating oocyte loss.

The ceRNA hypothesis provides a theoretical framework for understanding how lncRNAs may potentially regulate gene expression by competitively binding shared miRNAs [33]. Our computationally predicted network identified two candidate ceRNA axes with hypothetical roles in DOR: lncRNA MSTRG.39605.16/miR-143-5p/FADS1 and lncRNA NONHSAT208904.1/miR-199b-3p/ACP3. It is critical to emphasize that these proposed axes are supported solely by bioinformatic prediction and inverse co-expression correlation; direct competitive binding among these RNAs has not been experimentally demonstrated.

FADS1 (fatty acid desaturase 1) encodes delta-5 desaturase, a rate-limiting enzyme in polyunsaturated fatty acid biosynthesis [34]. FADS1 has been implicated in various metabolic disorders and cancers, with recent evidence suggesting its involvement in AKT-mTOR pathway activation [35]. MiR-143-5p has been reported to inhibit AKT/mTOR signaling in multiple cell types [36].Our data showing downregulated miR-143-5p and upregulated FADS1 in young DOR patients are consistent with the hypothetical model that lncRNA MSTRG.39605.16 might potentially function as a miR-143-5p sponge. This predicted interaction would potentially derepress FADS1 expression, thereby altering fatty acid composition and cell signaling in granulosa cells. However, this interpretation is speculative and requires direct experimental validation, such as luciferase reporter assays and RNA immunoprecipitation, to confirm the proposed competitive binding relationships.

ACP3 (acyl carrier protein 3) is involved in fatty acid synthesis as an essential cofactor [37]. Although less characterized than other ACP family members, ACP3 likely participates in mitochondrial fatty acid synthesis and metabolic regulation. The upregulation of ACP3 and downregulation of its predicted regulator miR-199b-3p were observed in our DOR samples. These expression patterns, coupled with the predicted binding relationship of lncRNA NONHSAT208904.1, are compatible with a hypothetical model of metabolic dysregulation in GCs from young women with DOR.

Both validated ceRNA axes converge on fatty acid metabolism, reinforcing our pathway analysis results. This metabolic focus aligns with emerging evidence that ovarian aging and dysfunction are associated with altered lipid metabolism, mitochondrial dysfunction, and oxidative stress [38]. The hypothetical roles of these predicted ceRNA networks in DOR pathogenesis require rigorous functional validation, including direct interaction assays (e.g., luciferase reporter assays, RNA immunoprecipitation) and functional perturbation studies (e.g., gene knockdown/overexpression) in appropriate in vitro and in vivo models.

## Limitations and future directions

This study has several limitations. First, the sample size for sequencing was relatively small (*n* = 4 per group), which raises legitimate concerns regarding statistical power, reproducibility, and false discovery rate inflation. This sample size was constrained by strict quality control requirements (RIN ≥ 6.0) and the limited availability of clinically well-characterized granulosa cell samples from young DOR patients. Nonetheless, this sample size is consistent with several published ceRNA and transcriptomic studies in granulosa cells [39, 40]. To partially mitigate this limitation, we validated key findings in an independent cohort (*n* = 28 per group) using qRT-PCR (Fig. 6), and results from both the discovery and validation cohorts were concordant. Second, our cross-sectional design cannot establish causality between the identified molecular alterations and DOR phenotype. Meanwhile, ovarian stimulation protocols (GnRH agonist vs. antagonist) were evenly distributed between groups, but we did not perform multivariate adjustment for stimulation regimen, gonadotropin dosage, or other clinical variables. Future studies with larger cohorts should incorporate multivariate modeling to control for these potential confounders. Third, functional validation of the ceRNA networks was not performed in this study; the proposed ceRNA axes therefore remain hypothetical.

Future research should employ granulosa cell-specific or oocyte-specific genetic manipulation in animal models to elucidate the causal roles of the identified ceRNA axes in ovarian reserve maintenance. Functional assays including luciferase reporter assays and RNA immunoprecipitation should be performed to confirm direct molecular interactions within the ceRNA networks. Additionally, longitudinal studies are needed to determine whether the identified molecular signatures can predict DOR development in young women with initially normal ovarian reserve markers, and multivariate modeling with larger cohorts is warranted to control for confounding clinical variables.

## Conclusion

In conclusion, this comprehensive transcriptome analysis of granulosa cells from young women with DOR reveals significant alterations in miRNA, lncRNA, and mRNA expression profiles, predominantly enriched in fatty acid metabolism, AKT-mTOR signaling, and apoptosis pathways. Acknowledging the limitations of a small sequencing discovery cohort (*n* = 4 per group) and the absence of functional validation, these findings should be interpreted with appropriate caution. The predicted ceRNA networks (lncRNA MSTRG.39605.16/miR-143-5p/FADS1 and lncRNA NONHSAT208904.1/miR-199b-3p/ACP3) provide hypothesis-generating insights into potential molecular mechanisms underlying DOR in young women and represent candidate targets for future mechanistic and therapeutic studies, pending comprehensive functional validation of the proposed direct interactions and biological effects.

## Supplementary Information

Supplementary Material 1: Fig. s1. Sequencing-based validation of differentially expressed miRNAs. Fig. s2. Sequencing-based validation of differentially expressed lncRNAs. Violin plots showing expression levels of five lncRNAs in YD and YN groups. Fig. s3. Sequencing-based validation of differentially expressed mRNAs.

## Data Availability

All data generated or analyzed during this study are included in this published article and its supplementary information files. Raw sequencing data are available from the corresponding author upon reasonable request.

## Abbreviations

DOR: Diminished ovarian reserve
GCs: Granulosa cells
miRNA: MicroRNA
lncRNA: Long non-coding RNA
mRNA: Messenger RNA
ceRNA: Competing endogenous RNA
NOR: Normal ovarian reserve
FSH: Follicle-stimulating hormone
AMH: Anti-Müllerian hormone
AFC: Antral follicle count
IVF-ET: In vitro fertilization-embryo transfer
GO: Gene Ontology
KEGG: Kyoto Encyclopedia of Genes and Genomes
qRT-PCR: Quantitative real-time PCR
BMI: Body mass index
Gn: Gonadotropin
FORT: Follicular output rate
MII: Metaphase II
2PN: Two pronuclei
FADS1: Tatty acid desaturase 1
ACP3: Acyl carrier protein 3
PCA: Principal component analysis
FDR: False discovery rate
RIN: RNA integrity number
TPM: Transcripts per million
FPKM: Fragments per kilobase of transcript per million mapped reads
MRE: MiRNA response element
3'-UTR: 3’-untranslated region
