# Supplementary material for: Transcriptome analysis of follicular granulosa cells in young women with diminished ovarian reserve
Source: Reprod Biol Endocrinol. 2026 Jul 15;24:94. doi: 10.1186/s12958-026-01589-y (PMC13651118; doi:10.1186/s12958-026-01589-y)
Supplement: Supplementary file 1 [file 12958_2026_1589_MOESM1_ESM.pdf]

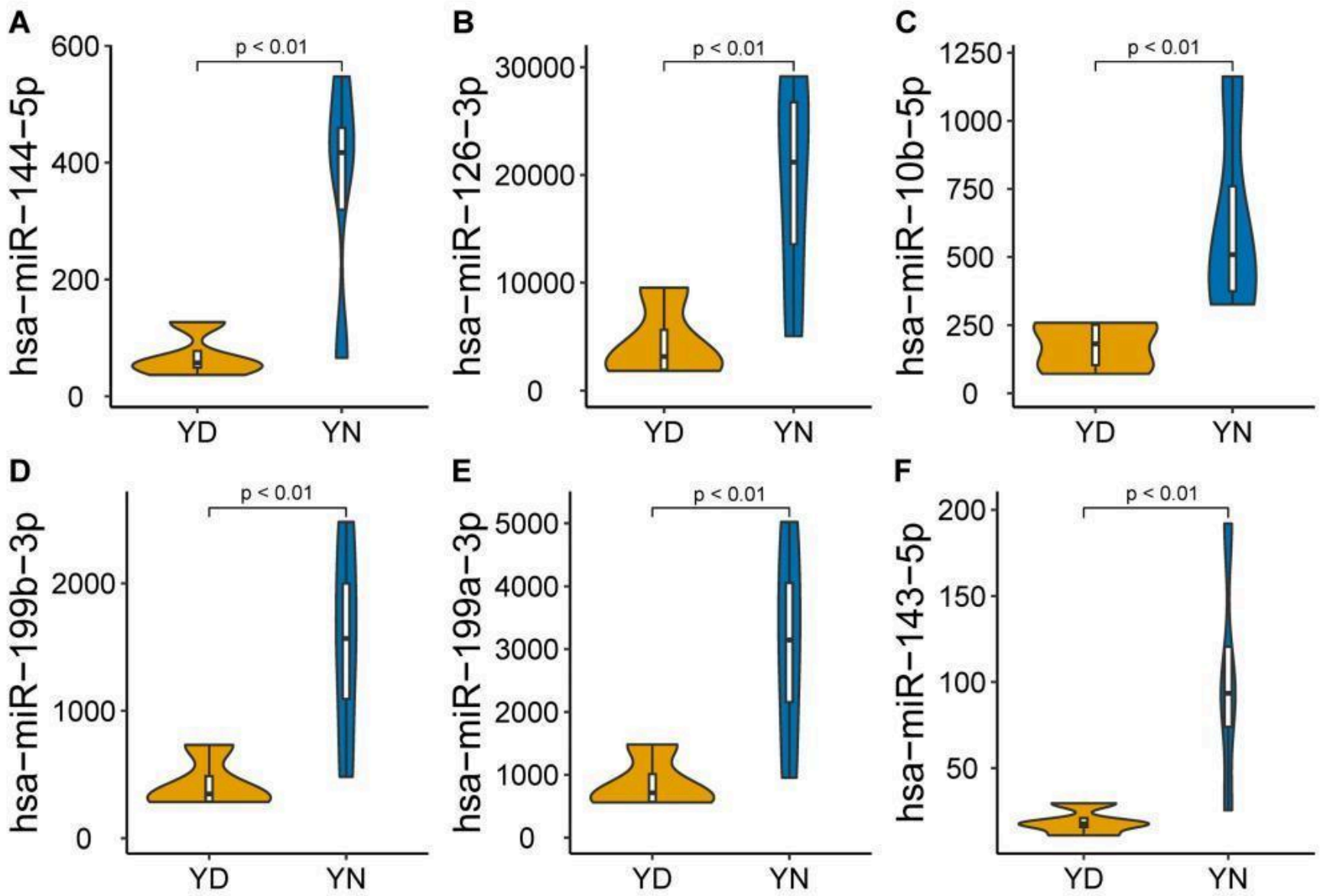

Fig. s1. Sequencing-based validation of differentially expressed miRNAs.

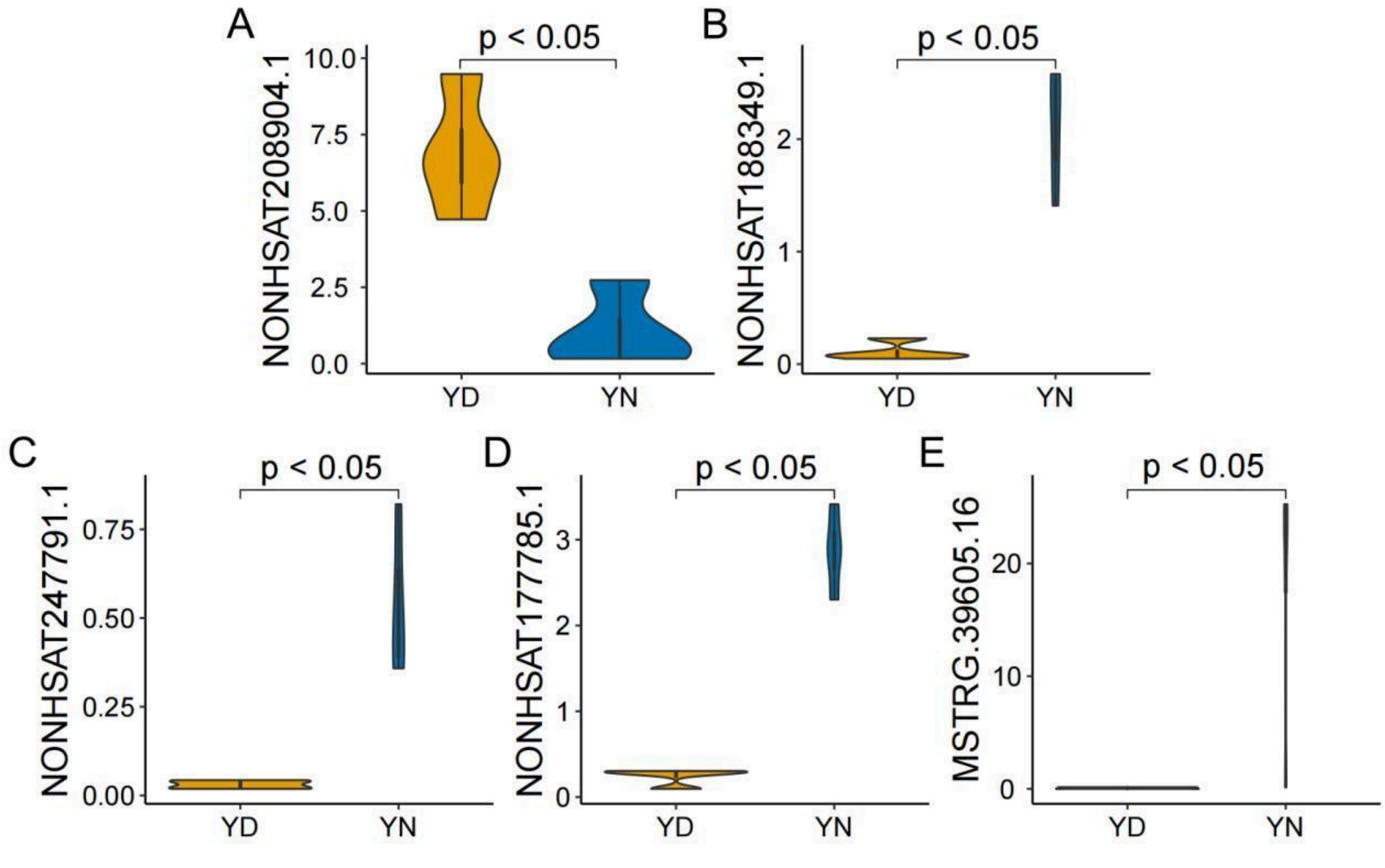

Fig. s2. Sequencing-based validation of differentially expressed lncRNAs. Violin plots showing expression levels of five lncRNAs in YD and YN groups.

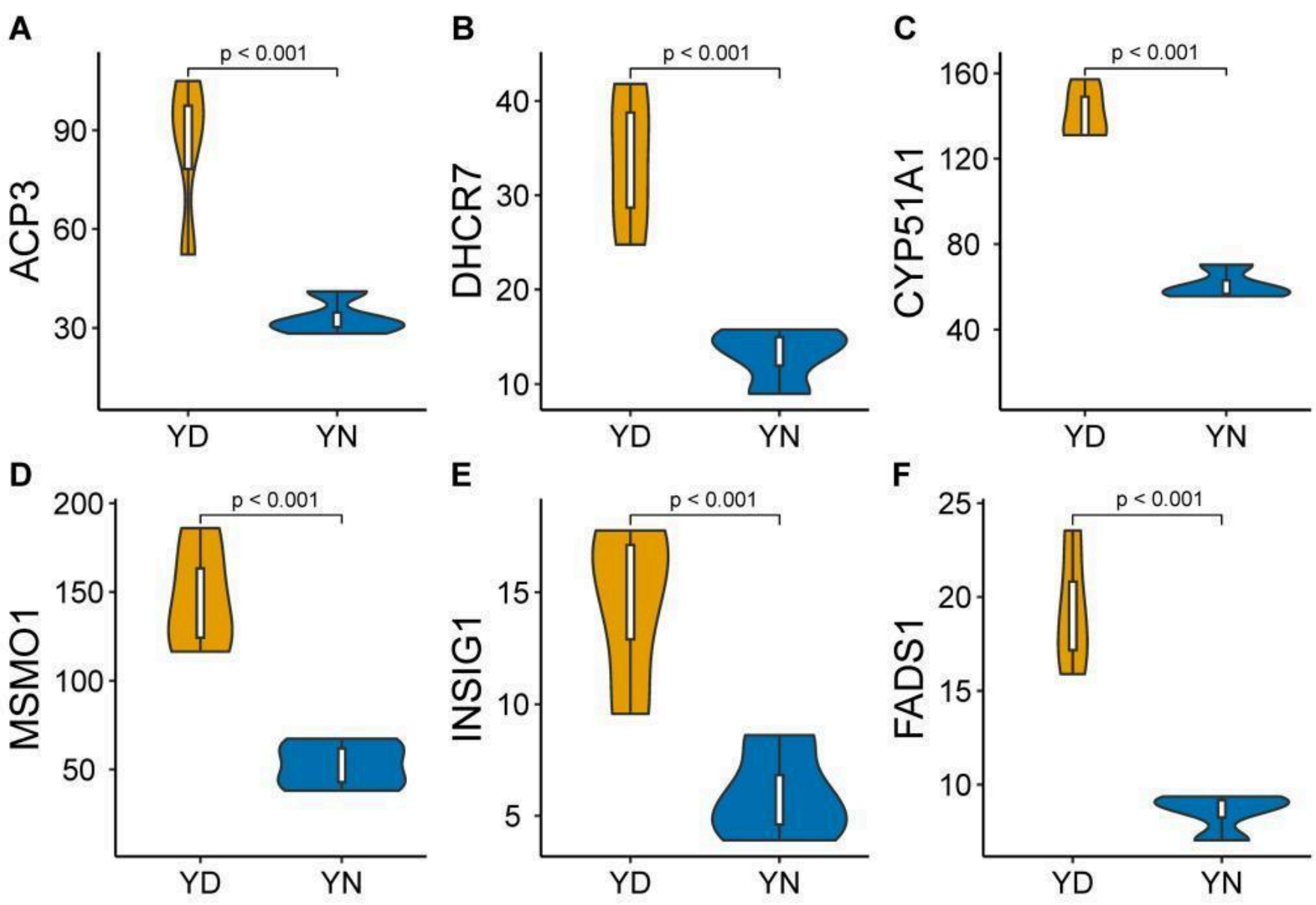

Fig. s3. Sequencing-based validation of differentially expressed mRNAs.
